# Oxidative Stress in Diabetic Retinopathy: A Comprehensive Review of Mechanisms, Biomarkers, and Therapeutic Perspectives

**DOI:** 10.3390/antiox14101204

**Published:** 2025-10-04

**Authors:** Tatsuya Mimura, Hidetaka Noma

**Affiliations:** 1 Department of Ophthalmology, School of Dental Medicine, Tsurumi University, Yokohama 230-0063, Japan; 2 Department of Ophthalmology, Tokyo Medical University Ibaraki Medical Center, Ami-cho 300-0395, Japan

**Keywords:** diabetic retinopathy, oxidative stress, reactive oxygen species (ROS), blood-retinal barrier (BRB), mitochondrial dysfunction, antioxidant therapy

## Abstract

Diabetic retinopathy (DR) is a leading cause of vision loss globally and represents one of the most common microvascular complications of diabetes. In addition to metabolic disturbances associated with hyperglycemia, oxidative stress has emerged as a critical contributor to the onset and progression of DR. Oxidative stress, defined as an imbalance between the production of reactive oxygen species (ROS) and antioxidant defense mechanisms, leads to cellular injury, inflammation, and increased vascular permeability. In the diabetic retina, excessive ROS production promotes endothelial cell apoptosis, breakdown of the blood-retinal barrier (BRB), and induction of angiogenic factors such as vascular endothelial growth factor (VEGF). This review provides a comprehensive overview of the pathophysiology of DR, focusing on the molecular mechanisms of oxidative stress. Relevant studies were identified through a structured search of PubMed, Web of Science, and Scopus (2000–2025) using terms such as ‘diabetic retinopathy’, ‘oxidative stress’, and ‘antioxidants’. We explore current knowledge on oxidative stress-related biomarkers and therapeutic strategies targeting oxidative damage, including antioxidant compounds and mitochondrial protective agents. Recent findings from both experimental and clinical studies are summarized, highlighting the translational potential of oxidative stress modulation in DR management. Finally, future research directions are discussed, including biomarker standardization, personalized medicine approaches, and long-term clinical validation of antioxidant-based therapies. A deeper understanding of oxidative stress may offer valuable insights into novel diagnostic and therapeutic strategies for DR.

## 1. Introduction

Diabetic retinopathy (DR) is the most common and one of the most serious microvascular complications associated with diabetes mellitus, and it remains a leading cause of vision loss globally [1]. According to the 11th edition of the International Diabetes Federation (IDF) Diabetes Atlas (2025), over 530 million people worldwide are living with diabetes, and approximately 30% of them are affected by some degree of DR [2]. Among the various forms of DR, proliferative DR (PDR) and diabetic macular edema (DME) are the major causes of significant visual impairment, imposing substantial socioeconomic burdens [3].

The pathophysiology of DR is multifactorial and progresses through multiple stages, involving complex interactions among hyperglycemia-induced metabolic dysregulation, chronic inflammation, endothelial dysfunction, and neurodegeneration [4]. Among these mechanisms, oxidative stress has recently gained increasing attention. Oxidative stress is defined as a condition in which the balance between the generation of reactive oxygen species (ROS) and antioxidant defense systems is disrupted, leading to cellular and tissue damage [5]. While ROS play essential roles in cellular metabolism and signaling under physiological conditions, excessive ROS production results in lipid peroxidation, DNA damage, and protein modification and ultimately contributes to cell death and inflammation.

In diabetes, several biochemical pathways contribute to ROS overproduction, including mitochondrial dysfunction, activation of the polyol pathway, accumulation of advanced glycation end products (AGEs), and enhanced activity of nicotinamide adenine dinucleotide phosphate (NADPH) oxidase. These alterations, along with impaired antioxidant capacity, create a chronic state of oxidative stress [6,7]. Such oxidative stress is implicated in endothelial injury within retinal capillaries, disruption of the blood–retinal barrier (BRB), and degeneration of retinal ganglion cells, thereby playing a central role in the development and progression of DR.

This review provides a comprehensive overview of the pathophysiological significance of oxidative stress in DR. We summarize current findings related to ROS-associated biomarkers, recent advances in experimental and clinical research, and emerging therapeutic strategies targeting oxidative stress. A deeper understanding of DR from the perspective of oxidative stress may lead to the development of novel therapeutic approaches and preventive strategies in the future.

## 2. Method and Literature Search Strategy

This article is a narrative review. We conducted a comprehensive literature search using the PubMed, Web of Science, and Scopus databases to identify relevant studies published between January 2000 and August 2025. The search strategy combined the following keywords and MeSH terms: “*diabetic retinopathy*”, “*oxidative stress*”, “*reactive oxygen species*”, “*antioxidants*”, and “*vascular endothelial growth factor*”. Only articles published in English were considered. Both original research (clinical, in vivo, and in vitro studies) and review articles that addressed the pathophysiology of oxidative stress, biomarkers, or therapeutic strategies in DR were included. Exclusion criteria were studies not directly related to DR, articles without sufficient methodological detail, and non–peer-reviewed sources. Additional references were identified by reviewing the bibliographies of selected articles to ensure comprehensive coverage. Although not conducted as a systematic review, this approach allowed for a comprehensive synthesis of the available evidence.

The initial search yielded approximately 982 records. After screening titles and abstracts for relevance, 142 articles were retained, and subsequent full-text review with application of predefined inclusion and exclusion criteria resulted in a final selection of 53 articles. From this pool, we specifically focused on studies and review articles that examined the relationship between DR and antioxidant mechanisms. These references were synthesized to provide a comprehensive overview of the molecular basis of oxidative stress in DR, as well as the potential of antioxidant-based therapeutic strategies.

## 3. Pathophysiology of DR

**Figure 1:**
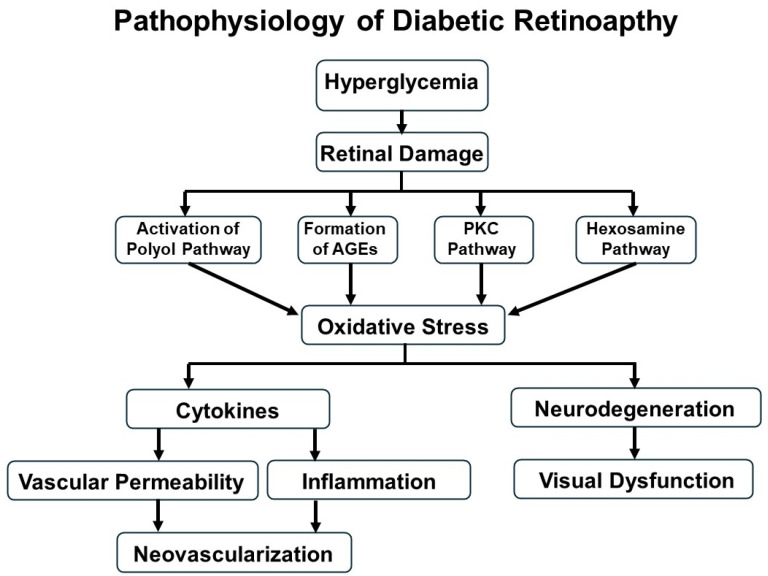
Pathophysiological mechanisms involved in DR.

DR is characterized by progressive retinal microvascular alterations and neurodegeneration resulting from chronic hyperglycemia-induced damage to the retinal microcirculation. DR is now recognized as a disease characterized by two major interrelated processes: retinal neurodegeneration and retinal vasculopathy. Accumulating evidence indicates that retinal neurodegeneration represents an early event that may precede clinically detectable vascular alterations, such as microaneurysm formation and capillary non-perfusion [8]. This dual-pathway perspective highlights the need to consider both neuronal and vascular targets when exploring the pathogenesis and treatment of DR. As illustrated in Figure 1, the pathogenesis and progression of DR involve a complex interplay of metabolic dysregulation, oxidative stress, inflammation, increased vascular permeability, and pathological angiogenesis [4].

### 3.1. Hyperglycemia-Induced Pathways and Retinal Damage

Persistent hyperglycemia triggers a cascade of harmful events in the diabetic retina. It activates several biochemical pathways, including the polyol pathway, AGE formation, protein kinase C (PKC) activation, and the hexosamine pathway flux [6]. These alterations impair retinal microvascular homeostasis by inducing pericyte loss, endothelial dysfunction, and thickening of the basement membrane, ultimately leading to breakdown of the BRB. A unifying feature of these pathways is the overproduction of ROS, which amplifies vascular injury and inflammatory signaling. In addition, hyperglycemia-driven mitochondrial dysfunction further accelerates ROS generation through excessive electron transport chain activity, resulting in oxidative DNA damage, impaired intracellular signaling, and apoptosis of retinal cells [7,9].

### 3.2. Vascular Hyperpermeability and Inflammation

Oxidative stress contributes to the disruption of tight junctions between endothelial cells that form the BRB, thereby increasing vascular permeability [10]. Vascular endothelial growth factor (VEGF), a key mediator in this process, compromises tight junction integrity and promotes retinal edema and plasma leakage [11]. In parallel, hyperglycemia and oxidative stress stimulate the expression of inflammatory cytokines such as interleukin-1β (IL-1β), tumor necrosis factor-alpha (TNF-α), and monocyte chemoattractant protein-1 (MCP-1), leading to leukocyte infiltration, microglial activation, and the establishment of a chronic inflammatory state in the retina. This inflammatory response is largely driven by activation of the nuclear factor kappa B (NF-κB) signaling pathway under oxidative conditions [12], further exacerbating endothelial dysfunction and enhancing the expression of angiogenic factors.

### 3.3. Angiogenesis

In the advanced stages of DR—particularly in PDR—retinal ischemia leads to the overexpression of angiogenic factors and the formation of abnormal and fragile neovessels. VEGF plays a central role in this process, with its gene expression being upregulated by hypoxia-inducible factor 1-alpha (HIF-1α), which is activated by both oxidative stress and inflammation [13]. These fragile new vessels are prone to vitreous hemorrhage and may cause tractional retinal detachment, significantly impairing vision.

ROS can drive upregulation of angiopoietin-2 (Ang-2) in diabetic retina through activation of NF-κB and AGE-RAGE signaling, contributing to pericyte loss and vascular destabilization [14]. Furthermore, ROS promote expression of galectin-3, which enhances neuroinflammation and glial activation in diabetic retina and contributes to neuronal damage and BRB dysfunction [15].

## 4. Biological Basis of Oxidative Stress

### 4.1. ROS and Antioxidant Defense Mechanisms

Oxidative stress refers to a pathological state in which the balance between the production of ROS and reactive nitrogen species (RNS), and the activity of antioxidant defense systems, is disrupted in favor of oxidative damage. ROS include superoxide anion (O_2_^−^), hydrogen peroxide (H_2_O_2_), and hydroxyl radicals (•OH), which are primarily generated by enzymatic systems such as mitochondria, NADPH oxidase, and xanthine oxidase [16]. Under physiological conditions, ROS play important roles in intracellular signaling and immune defense; however, excessive ROS can cause oxidative modifications of lipids, proteins, and nucleic acids, leading to cellular dysfunction and death.

To counteract these effects, cells are equipped with endogenous enzymatic antioxidants—such as superoxide dismutase (SOD), catalase (CAT), and glutathione peroxidase (GPx)—as well as non-enzymatic antioxidants, including vitamin C, vitamin E, and glutathione [17]. Among the sources of ROS, mitochondrial electron leakage from the electron transport chain plays a central role in the pathogenesis of DR. The retina, with its high oxygen consumption and intense metabolic activity, is particularly vulnerable to oxidative damage [7].

### 4.2. Mechanisms of Oxidative Stress–Induced Cellular Injury

Oxidative stress contributes to cellular injury through multiple mechanisms. Lipid peroxidation produces harmful by-products such as malondialdehyde (MDA) and 4-hydroxynonenal (4-HNE), which impair membrane fluidity and damage membrane proteins and receptors [18]. Oxidative DNA damage, marked by the accumulation of 8-hydroxy-2′-deoxyguanosine (8-OHdG), has been implicated in retinal cell apoptosis and mutagenesis.

Moreover, oxidative stress activates redox-sensitive transcription factors such as NF-κB and activator protein-1 (AP-1), leading to the upregulation of inflammatory cytokines (e.g., IL-6, TNF-α) and adhesion molecules (e.g., intercellular adhesion molecule-1 (ICAM-1)). These inflammatory responses contribute to endothelial dysfunction, breakdown of the BRB, and the development of retinal neovascularization and edema [19].

In addition, oxidative stress promotes neurodegeneration through activation of retinal glial cells—including Müller cells and microglia—and by impairing the function of retinal neurons. This reinforces the emerging concept that DR is not merely a vascular disorder but rather a disease of the neurovascular unit [20].

## 5. Role of Oxidative Stress in DR

### 5.1. Oxidative Stress and Capillary Damage

**Figure 2:**
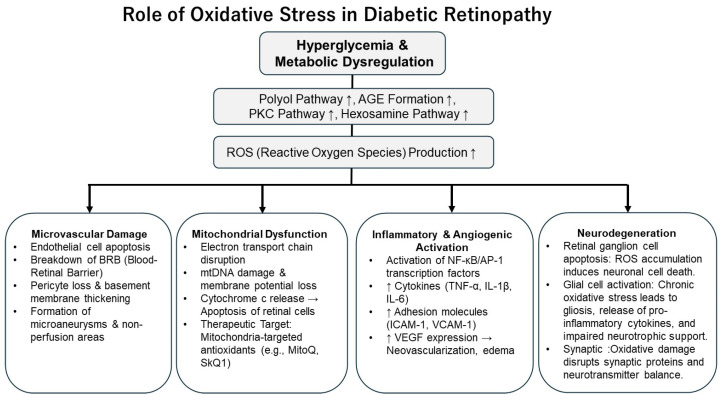
Schematic representation of the mechanisms by which chronic hyperglycemia contributes to oxidative stress in DR. Chronic hyperglycemia triggers metabolic pathways that elevate ROS levels, causing endothelial cell damage, disruption of the BRB, and formation of microaneurysms in DR. Arrows indicate changes in activity (↑, increased).

The early pathological features of DR include microvascular changes such as endothelial dysfunction, thickening of the basement membrane, and pericyte loss. Increasing evidence suggests that oxidative stress plays a pivotal role in the development of these abnormalities [7]. As illustrated in Figure 2, chronic hyperglycemia enhances glucose metabolism and activates pathways such as the polyol pathway and the formation of AGEs, leading to increased production of ROS. The resulting excess ROS induces apoptosis in retinal capillary endothelial cells, contributing to BRB breakdown and the formation of microaneurysms [21].

In retinal microvessels in particular, O_2_^−^–induced DNA damage and lipid peroxidation are critical mechanisms of cell death that exacerbate non-perfusion areas and promote retinal ischemia [16]. These oxidative insults are involved throughout the course of DR, from its earliest stages to more advanced disease.

### 5.2. Mitochondrial Dysfunction

The retina is a highly metabolically active tissue with high oxygen demand, making it especially vulnerable to mitochondrial ROS. Under hyperglycemic conditions, excessive ROS generation occurs due to dysregulation of the mitochondrial electron transport chain, leading to mitochondrial DNA (mtDNA) damage and a decrease in mitochondrial membrane potential [22]. These mitochondrial insults result in the activation of apoptotic pathways, such as cytochrome c release, and contribute to the death of retinal neurons and vascular endothelial cells.

Recent studies have demonstrated that the administration of mitochondria-targeted antioxidants such as MitoQ and SkQ1 significantly suppresses ROS generation and mitigates capillary damage in diabetic animal models, highlighting the potential of mitochondria as therapeutic targets in DR [23].

### 5.3. Induction of Inflammatory Cytokines and VEGF

Oxidative stress also activates redox-sensitive transcription factors such as NF-κB and AP-1, leading to increased expression of inflammatory cytokines (e.g., TNF-α, IL-1β, IL-6) and adhesion molecules (e.g., ICAM-1, vascular cell adhesion molecule-1 (VCAM-1)), thereby promoting chronic inflammation [18]. This inflammatory cascade enhances leukocyte adhesion to the vascular endothelium and increases vascular permeability, resulting in retinal edema and worsening of microvascular occlusion.

Furthermore, oxidative stress is deeply involved in the upregulation of VEGF, a key molecule that strongly promotes angiogenesis and vascular permeability. VEGF plays a central role in the pathogenesis of DME and PDR [24]. Given the centrality of anti-VEGF therapies in current DR treatment, targeting upstream oxidative stress mechanisms presents a promising strategy to suppress VEGF expression and disease progression at an earlier stage.

## 6. Oxidative Stress-Related Biomarkers

There is substantial evidence that oxidative stress plays a critical role in the onset and progression of DR [25]. The measurement of oxidative stress-related biomarkers is not only useful for understanding the underlying pathophysiology but also serves as a tool for assessing disease severity and monitoring therapeutic responses. In recent years, numerous studies have reported the quantification of oxidative stress-related molecules in clinical specimens such as blood, urine, and vitreous fluid.

### 6.1. Biomarker Profiles in Clinical Samples

Several representative markers reflect oxidative stress status, including 8-OHdG, MDA, advanced oxidation protein products (AOPP), isoprostanes, and total antioxidant capacity (TAC) [18]. Among these, 8-OHdG is a widely recognized indicator of oxidative DNA damage and has been shown to be elevated in the serum and urine of diabetic patients [26]. MDA, a final product of lipid peroxidation, is commonly measured in serum and has also been reported to correlate with the severity of DR based on serum levels [27]. AOPP and isoprostanes are typically assessed in serum or plasma, reflecting protein and lipid oxidation, respectively, whereas TAC is generally evaluated in serum or plasma as an integrated measure of systemic antioxidant defense.

In vitreous samples from patients with DR, increased levels of MDA and AOPP have been observed, along with decreased activities of antioxidant enzymes such as SOD and GPx, suggesting that oxidative stress is also locally elevated in the retina [7].

### 6.2. Correlation with Disease Progression

Multiple clinical studies have demonstrated significant associations between oxidative stress-related biomarkers and the progression of DR. For instance, Ng et al. reported that serum AOPP levels increased significantly as DR progressed from non-proliferative DR (NPDR) to PDR [28]. Furthermore, a meta-analysis indicated that levels of 8-OHdG and MDA were significantly higher in patients with DR compared to diabetic patients without retinopathy, supporting the potential utility of these biomarkers for monitoring disease progression [18].

Circulating lipid-peroxidation products such as MDA are generally elevated in patients with DR versus diabetic patients without retinopathy, as summarized in a recent systematic review and meta-analysis (higher pooled MDA; SMD 0.897). This body of evidence includes earlier clinical reports showing higher serum MDA in patients with retinopathy compared with diabetics without retinopathy [29].

Concurrently, several studies have documented altered antioxidant enzyme status with worsening DR. Lower activities of key antioxidant enzymes—notably SOD and CAT—have been observed in DR patients and correlate with retinopathy severity in cross-sectional cohorts [30]. Some multicenter and larger cross-sectional investigations demonstrated decreased SOD/GSH-Px or SOD/CAT in diabetes compared with controls, and more recent work with larger samples confirms that reduced SOD/CAT is associated with NPDR and PDR stages [31].

Mechanistically, mitochondrial dysfunction and impaired Mn-SOD (mitochondrial SOD) contribute to retinal oxidative damage and capillary cell apoptosis in experimental models, linking enzyme dysregulation to pathophysiological processes relevant to clinical progression [32].

Taken together, these findings support the potential utility of a panel including lipid-peroxidation markers (e.g., MDA/TBARS, isoprostanes) and antioxidant enzymes (SOD, CAT, GPx), plus DNA-oxidation markers (8-OHdG) for early detection, staging, and therapeutic monitoring of DR. However, heterogeneity in assay methods, small sample sizes in many studies, cross-sectional designs, and lack of universal reference ranges limit clinical translation. Prospective, well-powered cohort studies with standardized sampling and assay protocols are therefore required to (1) determine whether biomarker changes precede clinical progression, (2) establish clinically actionable thresholds, and (3) evaluate whether biomarker-guided antioxidant or other interventions alter DR trajectories [29,30,32].

## 7. Therapeutic Strategies Targeting Oxidative Stress

**Figure 3:**
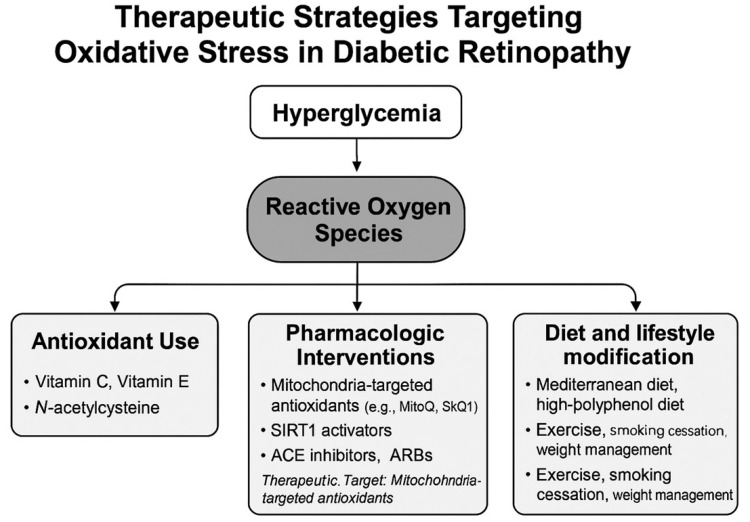
Overview of therapeutic strategies targeting oxidative stress in DR. NAC: N-Acetylcysteine, MitoQ: Mitoquinone, SkQ1: Skulachev Ion-1, SIRT1: Sirtuin 1 (Silent Information Regulator 1), ACE inhibitors: Angiotensin-Converting Enzyme inhibitors, ARBs: Angiotensin II Receptor Blockers, DR: Diabetic retinopathy, ROS: Reactive Oxygen Species.

Oxidative stress is a central pathogenic factor in the onset and progression of DR, and therapeutic approaches targeting oxidative stress have garnered increasing attention for both prevention and treatment. Figure 3 illustrates an overview of therapeutic strategies aimed at mitigating oxidative stress in DR. ROS induced by hyperglycemia contribute to retinal vascular injury, neurodegeneration, and inflammatory changes. Therefore, antioxidant-based therapies aimed at suppressing ROS generation offer a mechanistically grounded and promising treatment avenue [7].

### 7.1. Use of Antioxidants (Vitamin C, Vitamin E, N-acetylcysteine: NAC)

Among the antioxidant agents, vitamin C (ascorbic acid) and vitamin E (α-tocopherol) have been most extensively studied. Vitamin C, a water-soluble antioxidant, scavenges ROS in the bloodstream and within retinal cells, whereas vitamin E, a fat-soluble compound, inhibits lipid peroxidation in cell membranes. Experimental evidence from in vitro studies, animal models, and clinical trials has demonstrated that the combined administration of vitamins C and E can suppress the progression of retinal capillary damage in diabetic models [7,33].

NAC, a precursor of glutathione, enhances endogenous antioxidant capacity. In vivo studies in diabetic rat models have demonstrated that NAC attenuates retinal oxidative stress and suppresses the expression of inflammatory cytokines, thereby reducing retinal injury [26]. However, evidence from human clinical studies remains limited, and further investigation is required to validate its therapeutic potential in patients with DR [34].

### 7.2. Pharmacological Interventions: Mitochondria-Targeted Antioxidants and Novel Agents

Agents such as Mitoquinone (MitoQ) and Skulachev Ion-1 (SkQ1), which selectively accumulate in mitochondria, have demonstrated the ability to inhibit mitochondrial ROS production in rodent models of DR [35]. Similarly, compounds that activate SIRT1 have been shown in experimental mouse and rat models to promote mitochondrial biogenesis and reduce oxidative injury, thereby exerting neurovascular protective effects [36]. To date, however, evidence from human studies remains scarce, and further clinical trials are warranted to establish their therapeutic potential in patients with DR.

Angiotensin-converting enzyme (ACE) inhibitors and angiotensin II receptor blockers (ARBs) have demonstrated potential in slowing DR progression. Evidence from systematic reviews and meta-analyses indicates that their beneficial effects are mediated not only by lowering systemic blood pressure but also through mechanisms such as reducing retinal oxidative stress, attenuating inflammation, improving endothelial function, and decreasing vascular permeability [37]. These pleiotropic effects suggest that renin–angiotensin system inhibition may confer direct retinal protection beyond hemodynamic control.

### 7.3. Dietary and Lifestyle Modifications

Beyond pharmacotherapy, dietary and lifestyle interventions play an essential role in oxidative stress management. Mediterranean-style diets and polyphenol-rich diets have been associated with reduced oxidative and inflammatory stress, thereby lowering the risk of DR in patients with type 2 diabetes [38]. Specifically, plant-derived antioxidants such as flavonoids, resveratrol, and lutein have shown potential in protecting retinal tissues from oxidative injury.

In addition, regular physical activity, smoking cessation, and weight management are effective in reducing systemic inflammation and oxidative stress, while improving retinal circulation. A comprehensive strategy that incorporates lifestyle changes in conjunction with pharmacological therapy may offer synergistic benefits by lowering both systemic and local oxidative burdens [39].

## 8. Recent Advances in Experimental and Clinical Studies

The role of oxidative stress in the onset and progression of DR has been further elucidated through recent studies using animal models and human clinical investigations. Notably, interventions targeting oxidative stress have shown potential in preventing or ameliorating retinal damage, providing growing evidence to support future therapeutic strategies. Recent research findings are illustrated in Table 1 [9,40,41,42,43,44,45,46].

### 8.1. Experimental Studies Using Animal Models

Studies employing diabetic animal models—particularly streptozotocin (STZ)-induced diabetic rats and db/db mice—have consistently demonstrated elevated retinal oxidative stress markers such as MDA, 8-OHdG, and ROS production, alongside decreased antioxidant enzyme activities including SOD and GPx, even in the early stages of diabetes [47,48]. For instance, Kowluru and colleagues reported that administration of antioxidants such as α-lipoic acid and NAC to STZ-induced diabetic rats significantly inhibited retinal capillary degeneration and apoptosis [49].

Moreover, recent studies have shown that mitochondria-targeted antioxidants such as MitoQ and SkQ1 suppress ROS generation within retinal cells and contribute to the preservation of visual function [23]. These findings suggest that oxidative stress plays a critical role from the early phase of DR pathogenesis and represents a potentially reversible therapeutic target.

### 8.2. Findings from Human Interventional Studies

Although interventional trials targeting oxidative stress in human DR patients remain limited, the number of reports has been gradually increasing. For example, supplementation with antioxidant nutrients—such as vitamins C and E, lutein, and zeaxanthin—has been reported to help maintain visual function and retinal structure in patients with NPDR [50,51].

Additionally, lutein and zeaxanthin supplementation has been suggested to reduce retinal oxidative stress and improve visual performance, indicating a potential role for antioxidant therapy in clinical practice [52]. Furthermore, recent randomized controlled trials (RCTs) have reported that the intake of plant-derived antioxidants such as resveratrol significantly improves retinal blood flow and oxidative stress markers [53].

However, these effects vary considerably between individuals, and outcomes are influenced by factors such as dosage, duration of intervention, and concurrent treatments. Therefore, further validation through large-scale prospective studies is essential to establish standardized protocols and clinical efficacy.

## 9. Future Directions and Perspectives

Although the involvement of oxidative stress in the pathogenesis of DR has been strongly supported by both experimental and clinical studies, several important challenges remain to be addressed before these findings can be fully translated into clinical practice.

First, standardization in the measurement and evaluation of oxidative stress-related biomarkers is urgently needed. Currently, candidates such as MDA, 8-OHdG, and TAC have been proposed; however, discrepancies in sample types (e.g., blood, urine, vitreous humor), analytical methods, and cutoff values hinder their consistent application in clinical settings [16,18]. Establishing a reliable panel of biomarkers would greatly enhance the accuracy of disease staging and the monitoring of therapeutic efficacy in DR.

Second, the incorporation of oxidative stress into precision medicine frameworks represents a promising future direction. For instance, genetic polymorphisms in oxidative stress-related genes such as SOD2 and NFE2L2 have been shown to influence individual susceptibility to oxidative damage and response to antioxidant therapy [54,55]. Personalized oxidative stress-targeted interventions, based on genomic and metabolic profiling, could offer tailored and more effective therapeutic strategies for DR patients.

Third, many studies on oxidative stress interventions have been limited to short-term and small-scale trials. There is a lack of robust data regarding the long-term efficacy and safety of such treatments. In particular, for emerging therapies such as mitochondria-targeted antioxidants and plant-derived compounds, large-scale, multicenter, RCTs are essential to validate their clinical utility and risk–benefit profiles [23,24].

Beyond current findings, oxidative stress-related biomarkers such as MDA, 8-OHdG, and AOPP could be developed into standardized diagnostic panels, improving early detection and monitoring of DR progression. On the therapeutic side, novel antioxidant strategies—including mitochondria-targeted agents (e.g., MitoQ, SkQ1), Nrf2 activators, and natural compounds like curcumin—hold promise for clinical application. Furthermore, integration of genetic and metabolic profiling could facilitate precision medicine approaches, tailoring antioxidant interventions to individual patient risk and responsiveness.

Addressing these challenges will require stronger collaboration between basic and clinical researchers. The development of retina-specific oxidative stress evaluation systems and the accumulation of high-quality evidence for novel therapeutic strategies will be critical in moving the field forward.

## 10. Limitations

This review is limited by the heterogeneity of the included studies, ranging from preclinical models to small-scale clinical trials, which makes direct comparison challenging. Many antioxidant therapies remain in early phases of investigation, and long-term clinical evidence is scarce. Furthermore, publication bias and variability in biomarker measurement methods may have influenced the available evidence. These limitations underscore the need for standardized protocols and large-scale, longitudinal studies to confirm the therapeutic relevance of oxidative stress modulation in DR.

## 11. Discussion

Oxidative stress is increasingly recognized as a pivotal mechanism underlying the development and progression of DR. Evidence from both experimental and clinical studies supports its role in endothelial dysfunction, breakdown of the BRB, and promotion of angiogenesis through VEGF upregulation. Antioxidant strategies, including dietary compounds and mitochondrial protective agents, show promise in mitigating oxidative damage, although their clinical efficacy remains inconsistent. Translational efforts highlight the importance of integrating oxidative stress modulation into comprehensive management of DR. Moreover, the identification and validation of oxidative stress-related biomarkers may facilitate personalized treatment strategies in the future.

## 12. Conclusions

Oxidative stress plays a central role in the pathogenesis and progression of DR, contributing to multifactorial damage such as vascular dysfunction, chronic inflammation, and neurodegeneration within the retina. Numerous animal studies have demonstrated that both endogenous and exogenous antioxidant interventions can mitigate the development of retinal damage. Additionally, clinical studies have suggested that supplementation with antioxidants—including vitamins and polyphenols—may help reduce oxidative stress and preserve visual function.

However, several limitations currently hinder the widespread clinical application of oxidative stress-targeted therapies. These include a lack of standardization in biomarker assessment, significant inter-individual variability in treatment responses, and insufficient data regarding the long-term efficacy and safety of such interventions.

Future research should focus on the development of standardized evaluation systems and the integration of precision medicine approaches. These strategies will be essential for establishing effective, personalized therapeutic interventions targeting oxidative stress in DR.

## Figures and Tables

**Table 1:** Comparison between animal and clinical studies on oxidative stress interventions in DR [9,40,41,42,43,44,45,46].

Category	Animal Model Studies	Clinical Intervention Studies
Representative Models/Subjects	STZ-induced diabetic rats, db/db mice, transgenic models [8,40,41], Akita mouse [42]	NPDR/PDR patients with type 1 or type 2 diabetes [43,44,45]
Oxidative Stress Markers	↑ ROS, MDA, 8-OHdG, ↓ SOD/GPx, mitochondrial dysfunction [9,40,41]	Plasma oxidative balance score (OBS), retinal oxidative biomarkers [44,45]
Interventions	NAC, α-lipoic acid, MitoQ, SkQ1, SHP2 inhibition, NOX4 inhibitors [9,38,41,46]	Antioxidant supplements (vitamin C/E, lutein, resveratrol), polyphenols [43,44,45]
Key Outcomes	↓ Retinal apoptosis, ↓ inflammation, preserved visual function [9,40,41,46]	↓ Retinopathy progression, improved retinal perfusion, ↓ oxidative markers [43,44,45]
Mechanistic Insights	Mitochondrial ROS as central driver; SHP2-YAP1 axis; NOX4-mediated endothelial damage [41,46]	OBS inversely correlated with retinopathy severity and mortality [44]
Limitations	Species differences; translation to human pathology; short-term endpoints	Small sample sizes; variability in antioxidant bioavailability; need for RCTs

STZ: Streptozotocin, db/db mice: Diabetic BKS.Cg-Dock7^m +/+ Lepr^db/J Mice, ROS: Reactive Oxygen Species, MDA: Malondialdehyde, 8-OHdG: 8-Hydroxy-2′-deoxyguanosine, SOD: Superoxide Dismutase, GPx: Glutathione Peroxidase, NAC: N-Acetylcysteine, MitoQ: Mitoquinone, SkQ1: Skulachev Ion-1, SHP2: Src Homology Region 2-Containing Protein Tyrosine Phosphatase, NOX4: NADPH Oxidase 4, NPDR: Non-Proliferative DR, PDR: Proliferative DR, OBS: Oxidative Balance Score, RCT: Randomized Controlled Trial. Arrows indicate changes: ↑, increase; ↓, decrease.

## Data Availability

No new data were created or analyzed in this study. Data sharing is not applicable to this article.

## Abbreviations

The following abbreviations are used in this manuscript:

•OH	Hydroxyl radicals
4-HNE	4-hydroxynonenal
8-OHdG	8-hydroxy-2′-deoxyguanosine
ACE	Angiotensin-Converting Enzyme
AGE	Advanced glycation end product
AOPP	Advanced oxidation protein products
AP-1	Activator protein-1
ARBs	Angiotensin II Receptor Blockers
BRB	Blood-retinal barrier
CAT	Catalase
DME	Diabetic macular edema
GPx	Glutathione peroxidase
H_2_O_2_	Hydrogen peroxide
HIF-1α	Hypoxia-inducible factor 1-alpha
ICAM-1	Intercellular adhesion molecule-1
IDF	International Diabetes Federation
IL-1β	Interleukin-1β
MCP-1	Monocyte chemoattractant protein-1
MDA	Malondialdehyde
MitoQ	Mitoquinone
mtDNA	Mitochondrial DNA
NAC	N-Acetylcysteine,
NADPH	Nicotinamide adenine dinucleotide phosphate
NF-κB	Nuclear factor kappa B
NOX4	NADPH Oxidase 4
NPDR	Non-proliferative diabetic retinopathy
O_2_^−^	Superoxide anion
OBS	Oxidative balance score
PDR	Proliferative diabetic retinopathy
PKC	Protein kinase C
RCT	Randomized controlled trial
RNS	Reactive nitrogen species
ROS	Reactive oxygen species
SHP2	Src Homology Region 2-Containing Protein Tyrosine Phosphatase
SIRT1	Sirtuin 1 (Silent Information Regulator 1)
SkQ1	Skulachev Ion-1
SOD	Superoxide dismutase
STZ	Streptozotocin
TAC	Total antioxidant capacity
TNF-α	Tumor necrosis factor-alpha
VCAM-1	Vascular cell adhesion molecule-1
VEGF	Vascular endothelial growth factor
